# Role of Nrf2 in inflammatory response in lung of mice exposed to zinc oxide nanoparticles

**DOI:** 10.1186/s12989-019-0328-y

**Published:** 2019-12-16

**Authors:** Radwa Sehsah, Wenting Wu, Sahoko Ichihara, Naozumi Hashimoto, Yoshinori Hasegawa, Cai Zong, Ken Itoh, Masayuki Yamamoto, Ahmed Ali Elsayed, Soheir El-Bestar, Emily Kamel, Gaku Ichihara

**Affiliations:** 1 Department of Occupational and Environmental Health, Nagoya University Graduate School of Medicine, Nagoya, Japan; 2 Department of Environmental and Preventive Medicine, School of Medicine, Jichi Medical University, Shimotsuke, Japan; 3 Department of Respiratory Medicine, Nagoya University Graduate School of Medicine, Nagoya, Japan; 4 Department of Occupational and Environmental Health, Faculty of Pharmaceutical Sciences, Tokyo University of Science, Noda, Japan; 5 Department of Stress Response Science, Hirosaki University Graduate School of Medicine, Hirosaki, Japan; 6 Department of Medical Biochemistry, Tohoku University Graduate School of Medicine, Sendai, Japan; 7 Department of Pathology, Mansoura Faculty of Medicine, Mansoura, Egypt; 8 Department of Public Health and Community Medicine, Mansoura Faculty of Medicine, Mansoura, Egypt

**Keywords:** Oxidative stress, Pulmonary inflammation, Nrf2, Zinc oxide nanoparticles

## Abstract

**Background:**

Zinc oxide nanoparticles (ZnO-NPs) are widely used in many industrial sectors and previous studies have reported that exposure of the lungs to ZnO-NPs induces both acute and/or chronic pulmonary inflammation, but the exact mechanism underlying such response remains elusive. This study investigated the role of nuclear factor-erythroid 2-related factor (Nrf2) in pulmonary inflammation induced by exposure to ZnO-NPs using Nrf2 null (*Nrf2*^−/−^) mice.

**Methods:**

Twenty-four male *Nrf2*^−/−^ mice and thirty male wild type C57BL/6 J mice were divided into three groups of eight and ten each respectively, and exposed once to ZnO-NPs at 0, 10, 30 μg/mouse by pharyngeal aspiration. At 14 days after the exposure to ZnO-NPs, bronchoalveolar lavage fluid (BALF) and lungs were collected to quantify protein level and the number of inflammatory cells. The mRNA levels of *Nrf2*-dependent antioxidant enzymes and inflammatory cytokines in lung tissue were measured.

**Results:**

Exposure to ZnO-NPs dose-dependently increased the number of total cells, macrophages, lymphocytes and eosinophils in BALF both in *Nrf2*^−/−^ mice and wild type mice, but the magnitude of increase was significantly higher in *Nrf2*^−/−^ mice than wild type mice. The number of neutrophils in BALF increased in *Nrf2*^−/−^ mice, being accompanied by marginal trend of increase in mRNA expression of *MIP-2*, neutrophil chemoattractant, but such changes were not observed in wild type mice. Exposure to ZnO-NPs did not dose-dependently increase mRNA level of *Nrf2*-dependent antioxidant enzymes both in *Nrf2*^−/−^ mice and wild type mice.

**Conclusion:**

Pharyngeal aspiration of ZnO-NPs induced infiltration of inflammatory cells in the lung of mice, but minimally induced *Nrf2*-dependent antioxidant enzymes. The results suggest that *Nrf2* play a role in negative regulation on ZnO-NP exposure-induced neutrophil migration, but does not demonstrate that the regulation is through suppression of oxidative stress.

## Introduction

Engineered nanomaterials (ENMs) with their unique properties currently form a substantial part of many important industrial sectors, consumer products, as well as medicine [1]. Metal-based nanomaterials constitute a major category of ENMs and among them zinc oxide has received great attention and is one of the most commonly used nanomaterials due to its unique physical and chemical properties [2]. Zinc oxide nanoparticles (ZnO-NPs) are currently used in sunscreens, cosmetics, ceramics, paints, food packaging, solar cells and electronics [3].

The increased production and use of ZnO-NPs is expected to increase the potential of exposure, which might result in health ill-effects. Several groups have focused on the potential toxicity of ZnO-NPs but the results are inconsistent. Since the lungs are the main portal of entry into the human body for engineered nanomaterials in occupational settings [4], many studies have focused on the pulmonary effects of ZnO-NP exposure. Several in vitro studies showed high toxicity of ZnO-NPs in cell lines derived from pulmonary alveolar epithelial cells or macrophages [4–8]. Furthermore, in vivo studies showed that pulmonary exposure to ZnO-NPs produced bronchocentric interstitial pulmonary fibrosis [9], eosinophilic/fibrotic/granulomatous inflammation [10]. On the other hand, other studies showed that ZnO-NPs produced only short-term lung inflammation [11], minimal pulmonary inflammation [12], or transient pulmonary inflammation and cytotoxicity [13]. Similarly, the results of mechanistic studies on ZnO-NPs have been controversial. While many in vitro and in vivo studies provided strong evidence for the production of reactive oxygen species (ROS) and oxidative stress as the main mechanism of ZnO-NPs-induced cytotoxicity [14–17], Song et al. [18] stated that ROS was not necessarily the main contributing factor of ZnO-NPs-related cytotoxicity and Shen et al. [19] suggested that ROS may be the result of cytotoxicity rather than the cause. Other studies suggested that ZnO-NPs dissolution and release of zinc ion (Zn^2+^) are responsible for the observed cytotoxicity [9, 18, 20], which is supported by decreased dissolution and cytotoxicity of iron-doped ZnO-NPs [21]. In contrast, Cho et al. [22] showed that although the soluble component of ZnO-NPs was active in all pro-inflammatory assays in vitro, it had only modest potency in vivo, and Lin et al. [23] concluded that Zn^2+^ is not the cause of ZnO-NP cytotoxicity.

Since oxidative stress is the most widely accepted mechanism of nanoparticles-induced cytotoxicity [24], Nel and colleagues provided a hierarchical oxidative stress hypothesis; where at the lowest level of oxidative stress, nuclear factor erythroid 2-related factor 2 (NFE2L2, Nrf2) mediates the induction of antioxidant and detoxification enzymes (tier 1) [25]. At higher level of oxidative stress, the protective response is switched to inflammation through mitogen-activated protein kinases (MAPK) and nuclear factor kappa (NF-κB) cascades (tier 2). Finally, at the highest level of oxidative stress, apoptosis results from mitochondrial perturbation (tier 3).

*Nrf2* is a key transcription factor that regulates the antioxidant defense response against the harmful effects of ROS. Under normal conditions, *Nrf2* is sequestered in the cytoplasm by the regulatory protein Kelch-like ECH-associated protein 1 (Keap1). Under oxidative stress conditions, Nrf2 is released from Keap1 and translocated to the nucleus where it is heterodimerized with small Mafs, binds to the antioxidant response element (ARE) and finally up-regulates a group of antioxidant enzymes [26]. Functional polymorphisms in the promoter of *Nrf2* in humans are known to increase the risk of acute lung injury [27]. *Nrf2*^−/−^ mice have been developed to serve as a valuable model to examine Nrf2 pathway, and recently this model has been used to test the involvement and protective effect of Nrf2 in several lung diseases [28]. Indeed, *Nrf2*^−/−^ mice are found to be more susceptible to acute respiratory distress syndrome [29], asthma [30], emphysema [31], pulmonary fibrosis [32], ozone-related airway toxicity [33] and diesel exhaust particles [34].

The present study investigated the role of Nrf2 pathway and the potential role of oxidative stress in ZnO-NPs-induced pulmonary inflammation, using *Nrf2*^−/−^ mice model.

## Materials and methods

### Zinc oxide nanoparticles

ZnO-NPs (MKN-ZnO-020) with primary diameter of 20 nm were purchased from mkNano (Missisauga, ONT, Canada). The surface area was measured by Brunauer-Emmett-Teller (BET) (Macsorb HM model-1201, MOUNTECH, Tokyo, Japan). Endotoxin analysis was conducted using Pierce LAL Chromogenic Endotoxin Quantification Kit (Thermo Scientific, Waltham, MA). A biocompatible dispersion medium (DM) was used to disperse the nanoparticles, which was Ca^2+^- and Mg^2+^-free phosphate buffered saline (PBS, pH 7.4), supplemented with 5.5 mM D-glucose, 0.6 mg/ml mouse serum albumin, and 0.01 mg/ml 1, 2- dipalmitoyl-sn-glycero-3-phosphocholine (DPPC) [35, 36]. The nanoparticles were dispersed in the DM to prepare a suspension with a concentration of 0.25 μg NP/ml DM for the low-dose group (10 μg/mouse), another with a concentration of 0.75 NP μg/ml DM for the high-dose group (30 μg/mouse), and a suspension with only DM, without nanoparticles, for the control group. The particles were dispersed using a cup-type sonicator (Branson Sonifier, cup horn), at 100 W, 80% pulse mode for 10 min. The hydrodynamic size was determined using dynamic light scattering (DLS) (Zetasizer Nano-S; Malvern Instruments, Worcestershire, UK).

### Animals

*Nrf2*^−/−^ mice were produced as described by Itoh et al. [37] and backcrossed six times by the Central Institute for Experimental Animals and then further backcrossed seven times in Division of Experimental Animals, Nagoya University Graduate School of Medicine. The genotypes of mice were confirmed by PCR amplification of genomic DNA isolated from the tail. PCR amplification was carried out using three different primers, 5#-TGGACGGGACTATTGAAGGCTG-3# (Nrf2-sense for both genotypes), 3#-GCCGCCTTTTCAGTAGATGGAGG-5# (Nrf2-antisense for wild type), and 5#-GCGGATTGACCGTAATGGGATAGG-3# (Nrf2-antisense for LacZ). Another 24 pathogen-free age-matched male C57BL/6JJcl mice (*Nrf2*^+/+^) weighing 22–27 g were purchased from CLEA Japan Inc. (Tokyo). All mice were housed and acclimatized in a clean environment for 1 week before the start of exposure. Food and water were provided ad libitum. The animal room was light- and temperature-controlled with a 12-h light-dark cycle (lights on at 9 am and off at 9 pm), room temperature of 23–25 °C and relative humidity at 57–60%. One day before the exposure, all mice of both genotypes, were weighed and randomly divided into three groups (*n* = 8 in each group for *Nrf2*^−/−^ mice and *n* = 10 in each group for wild type mice); the control (0 μg ZnO-NPs), low-dose (10 μg ZnO-NPs) and high-dose (30 μg ZnO-NPs) groups. The guide of Japanese law concerning protection and control of animals and the guide of animal experimentation of Nagoya University School of Medicine were followed throughout the experiments. The animal experiment protocol was approved by Nagoya University Animal Experiment Committee.

### Pharyngeal aspiration of ZnO-NPs

Pharyngeal or oropharyngeal aspiration is proved to be an effective convenient alternative to inhalation exposure for the hazard assessment of nanomaterials [38]. The mouse was anesthetized by intraperitoneal injection of pentobarbital, and suspended by upper incisors encircled with a rubber band and its back was placed against an inclined board. Before administration, ZnO-NP suspensions were vortexed for 10 s. The tongue was gently extended by blunt forceps outside the oral cavity, and 40 μl aliquot of the selected concentration was pipetted into the back of the tongue, which was pulled for 1 min after pipetting then released. With the tongue protruded, the mouse was unable to swallow, and the liquid was aspirated into the lungs. Following release of the tongue, the mouse was gently lifted off the board, placed on its left side, and monitored for recovery from anesthesia. Mice of both genotypes received either DM, 10 or 30 μg ZnO-NPs, which were comparable to 0.5 or 1.5 mg/kg body weight. The lower concentration of 0.5 mg/kg is comparable to deposition of 0.48 mg/kg in adult human lung from inhalation to ZnO for one week at the threshold limit value of 2 mg/m^3^ (time-weighted average) proposed by American Conference of Governmental Industrial Hygienists (ACGIH), given the values of 500 mL air/breath, 12 breath/min, 40 h/week [39].

### Bronchoalveolar lavage (BAL), total and differential cell count

Fourteen days after exposure, mice were euthanized by intraperitoneal injection of pentobarbital. The trachea and lungs of each mouse were exposed and bronchoalveolar lavage was conducted. For this purpose, an 18-gauge needle was inserted into the trachea and both lungs were lavaged by 1 ml of 10% PBS (gentle instillation and aspiration). The instillation and aspiration of PBS was repeated 5 times, making a total volume of 5 ml. The amount of recovered bronchoalveolar lavage fluid (BALF) was measured and recorded. The average volume retrieved was > 90% of the 5 ml that was instilled, the amounts and the recovery rates were not different among the exposure groups. The collected BALF were kept on ice until centrifuged at 1500 rpm for 5 min, and the supernatant was aliquoted into three tubes and kept at − 80 °C until further analysis. The cell pellets were re-suspended in 1 ml of ACK lysis buffer (for red blood cells lysis) and left for 5 min at room temperature. Then 10 ml of 10% PBS were added and the whole volume was re-centrifuged at 1500 rpm for 5 min. The supernatant was discarded, and the cell pellet was re-suspended in 1 ml 10% PBS and kept on ice for use in total and differential cell counts. Total cell count was determined using a ChemoMetec Nucleocounter (Allerød, Denmark), while differential cell count was performed under optical microscope on slides prepared by cytospin and stained with May-Grunwald-Giemsa (Merck, Darmstadt, Germany). The cell types in the BALF included macrophages, neutrophils, lymphocytes and eosinophils. The relative differential counts were presented as percentages of total cells on 10 fields of each cytospin smear. The absolute differential count was calculated as the product of the number of the total cell count and the proportion of the relative differential count.

### Histopathological examination of the lung

After completion of BAL, the lungs were removed, washed in saline and the right lung was immediately frozen for further analysis. The left lung was fixed in 4% formalin, gradually dehydrated, embedded in paraffin, cut into 3 μm thick sections, placed on slides, stained with hematoxylin and eosin (H&E) and examined under optical microscope by a pathologist blinded to exposure. These lung sections were used to determine the degree of lung inflammation. The degree of peribronchial and perivascular inflammation was evaluated on a subjective scale of 0–3, as described previously [40, 41]. A score of 0 represented no detectable inflammation, while score of 1 represented occasional cuffing with inflammatory cells. For score 2, most bronchi or vessels were surrounded by a thin layer (1–5 cells thick) of inflammatory cells. For score 3, most bronchi or vessels were surrounded by a thick layer (> 5 cells thick) of inflammatory cells. Total lung inflammation was defined as the average of the peribronchial and perivascular inflammation scores. Four lung sections per mouse were scored and inflammation scores were expressed as the mean value. Tissue slides were examined under an optical microscope (model DM750, Leica Microsystem, Wetzlar, Germany) and images were captured with Leica Application Suite V3 software.

### Measurement of total protein in BALF

Total protein in BALF was measured using a Bio-Rad protein assay kit and the instructions provided by the manufacturer (Bio-Rad Laboratories, Hercules, CA) with bovine serum albumin (BSA) as a standard.

### Quantification of total glutathione and oxidized glutathione

Frozen lung tissue was homogenized with 5 volumes (w/v) of cold 50 mM MES buffer (pH 6.01) containing 1 mM EDTA. The protein in the sample was denatured with equal volume of 0.1% metaphosphoric acid (Sigma-Aldrich) and mixed on a vortex mixer. The mixture was allowed to stand at room temperature for 5 min and centrifuged at 2000 g for 3 min. The supernatant (95 μl) was kept at − 20 °C until used for determination of total glutathione and oxidized glutathione (GSSG). First, 90 μl of supernatant was treated with 4.5 μl of 4 M triethanolamine (Sigma-Aldrich) solution and vortexed well before assay. For total reduced form of glutathione (GSH) analysis, 30 μl TEAM-treated sample was diluted 20-fold with MES buffer (pH 6.0) containing 2 mM EDTA. An aliquot (50 μl) of the diluted solution was added 150 μl freshly prepared assay cocktail, and assayed at 405 nm with a microplate reader (Gen5™ & Gen5 Secure, BioTek® Instruments, Inc.). For GSSG determination, 30 μl of TEAM-treated sample was diluted 10 times with MES buffer before derivatization with 2-vinylpyridine., Two μl of 1 M 2-vinylpyridine was added to 200 μl of diluted solution of every sample or GSSG standard in tube, and then the tubes were mixed on a vortex mixer and incubated for 1 h at room temperature. Total GSH and GSSG concentrations were calculated from a standard curve using GSSG (Cayman; 703,014) prepared according to the GSH assay kit (Cayman Chemical Company, Ann Arbor, MI; 703,002), and normalized versus protein concentration. Total GSH and GSSG were expressed as micromoles of GSH (or GSSG) per milligram of protein.

### MDA assay

The MDA assay (Life Science Specialties, LLC; NWK-MDA01) was performed according to the protocol supplied by the manufacturer. A 10% wt/vol homogenate was prepared from lung tissue in cold Assay Buffer (Phosphate buffer, pH 7.0 with EDTA). Absorbance was read at 532 nm using a PowerScan4 microplate reader (DS Pharma Medical Co., Japan) after reaction of the sample with thiobarbituric acid (TBA). Samples were analyzed in duplicate, and MDA level was expressed as micromoles of MDA per milligram of protein.

### RNA isolation and real-time quantitative reverse transcription-polymerase chain reaction (RT-PCR)

The mRNA expression level of Nrf2-dependent genes; superoxide dismutase 1 *(SOD1)*, *catalase*, glutamate-cysteine ligase catalytic subunit *(GcLc),*glutamate-cysteine ligase modifier subunit *(GcLm),* NAD(P) H quinone oxidoreductase *(NQO1),* heme-oxygenase 1 *(HO-1)* and glutathione reductase *(GR),* and metal-binding protein genes; metallothioneins *(MT-1 and MT-2),* which play roles in protecting against oxidative stress, and inflammatory cytokines; tumor necrosis factor alpha *(TNF-α)*, interferon gamma *(IFN-γ)*, transforming growth factor beta *(TGF-β)*, interleukin-6 (*IL-6*), interleukin-1beta *(IL-1β),* monocyte chemotactic protein-1 *(MCP-1)*, chemokine (C-X-C motif) ligand 1 *(CXCL1, KC)* and chemokine (C-X-C motif) ligand 2 *(CXCL2, MIP-2),* fibrosis-related gene: matrix metalloproteinase 2 *(MMP2)* were measured in lung tissues. About 15 mg of frozen lung tissue were homogenized and total RNA was extracted using ReliaPrep™ RNA Tissue Miniprep System, treated with DNase (Promega, WI) and kept at − 80 °C until used. Concentrations of RNAs were determined with a Nanodrop-1000 3.5.1 (Thermo Fisher Scientific, Waltham, MA). The quality of isolated RNA was assessed by calculating the A260/ A280 ratio to ensure values between 1.7 and 2.0. For complementary DNA (cDNA) synthesis, SuperScript III Reverse transcriptase kit (Life Technologies, Carlsbad, CA) was used. The collected cDNA was kept at − 30 °C until quantified by quantitative real time PCR (Mx3005P QRCP System, Agilent Technologies, Waldbronn, Germany). The mRNA expression levels were normalized to *β-actin* for each gene. Primers and probes were designed by Universal Probe Library Assay Design Center (Roche Diagnostics). Primers` sequences are shown in (Additional file 1: Table S1).

### Statistical analysis

Data were expressed as mean ± standard deviation. Comparisons between the control and exposure groups were tested using Dunnett’s multiple comparison method or following one-way ANOVA or Steel multiple comparison method following Kruskal Wallis nonparametric test in each genotype. Single regression analysis or ordinal logistic regression analysis on the exposure level of ZnO nanoparticles was applied in each genotype separately. Multiple regression analysis or multiple ordinal logistic regression analysis using dummy variables for genotype was applied to examine the effects of genotype and trend with exposure level. When the interaction between genotype and exposure level was not significant, multiple regression analysis or multiple ordinal logistic regression analysis on exposure level and genotype in a model without interaction was applied to test the effects of exposure level and genotype. Statistical analysis was performed using software JMP version 14 (SAS Institute, Cary, NC) and probability (*p*) value < 0.05 was considered statistically significant.

## Results

### Characterization of ZnO-NPs

The surface area of primary ZnO nanoparticles measured by the BET gas absorption technique was 50.72 m^2^/g. No endotoxin was detected when the particles were suspended in distilled water. Dynamic light scattering showed aggregation of the nanoparticles in the dispersion medium with an average hydrodynamic size of 151.6 ± 0.7 nm. The presence of nano-sized particles was confirmed in the medium: the numbers of particles of less than 91.28 and 105.7 nm were 31.2% ± 0.8 and 71.2% ± 1.7%, respectively; the volume of particles of less than 91.28 and 105.7 nm were 19.6% ± 0.8 and 54.6% ± 2.1%, respectively.

### Changes in body and lung weights

Following pharyngeal aspiration, body weight decreased in all mice, which recovered rapidly from the third to fourth day post-exposure in both the control and 10 μg ZnO-NP groups of both genotypes. On the other hand, the 30 μg ZnO-NP groups of both genotypes lost significant body weight in the first week and did not regain it until post-exposure day eight. Body weight and relative lung weight (lung weight in mg/body weight in g) measured at post-exposure day 14 are shown in Table 1. In both genotypes, body weight was lower in the ZnO-NP exposed groups compared to the control group while the relative lung weight increased in a dose dependent manner with significant difference in the 30 μg ZnO-NP exposed group compared to the corresponding genotype control. Multiple regression analyses showed no significant interaction of ZnO level and genotype for body weight, lung weight and relative lung weight. Effect of genotype was not significant for all of the above indices.

**Table 1:** Body and lung weight of mice at 14 days after exposure to zinc oxide nanoparticles by pharyngeal aspiration

		Amount of ZnO-NPs administered (μg)			Simple regression	Multiple regression		
	Genotype	0	10	30	Effect of ZnO-NPs	Interaction of ZnO-NPs and Nrf2 deletion	Effect of ZnO-NPs	Effect of Nrf2 deletion
Body weight (g)	Wild type *Nrf2*^−/−^	25.9 ± 2.0 26.7 ± 1.9	26.1 ± 1.5 26.4 ± 1.9	25.2 ± 1.6 25.9 ± 2.1	−0.027 (*p* = 0.29) − 0.026 (*p* = 0.42)	0.00057 (*p* = 0.97)	0.020 (*p* = 0.18)	0.36 (*p* = 0.22)
Lung weight (mg)	Wild type *Nrf2*^−/−^	327 ± 34 331 ± 37	352 ± 36 339 ± 36	369 ± 56 378 ± 25^a^	1.3 (*p* = 0.043) 1.6 (*p* = 0.0076)	0.00014(*p* = 0.75)	0.0014(*p* = 0.0011)	−8.3 × 10^− 5^(*p* = 0.99)
Ratio of lung weight to body weight (×10^−3^)	Wild type *Nrf2*^−/−^	12.6 ± 0.9 12.4 ± 1.1	13.5 ± 1.0 12.8 ± 1.1	14.6 ± 1.9* 14.7 ± 1.5*	0.066 (*p* = 0.0019) 0.077 (*p* = 0.0008)	−0.0057 (*p* = 0.68)	0.07 (*p* < 0.0001)	--0.14(*p* = 0.41)

Mean ± SD. ^*^ *p* < 0.05compared to the corresponding genotype control by Dunnett’s multiple comparison following ANOVA. Simple regression analysis in each genotype and multiple regression analysis in a model with interaction was conducted. As the interaction was not significant, multiple regression in a model without interaction was finally conducted to estimate effect of ZnO-NPs or Nrf2 deletion

### Changes in BALF cytology and total protein

At post-exposure day 14, Dunnett’s multiple comparison following ANOVA or Kruskal Wallis nonparametric test showed that exposure to ZnO-NPs increased significantly total cell, macrophage and lymphocyte at 30 μg per mouse in both genotypes, while it significantly increased neutrophils at 30 μg per mouse only in *Nrf2*^−/−^ mice (Table 2). Exposure to ZnO-NPs increased eosinophils at 10 and 30 μg per mouse in *Nrf2*^−/−^ mice but increased eosinophils only at 30 μg per mouse in wild type mice (Table 2). Multiple regression analyses or multiple ordinal logistic regression analysis did not show significant interaction of ZnO-NPs exposure level and *Nrf2* deletion for the above parameters, thus multiple regression model without the interaction was applied for them, although marginally significant interaction is noted for neutrophils (*p* = 0.06) as it indicates different effect of ZnO-NP exposure level depending on the genotype. The results showed significant positive effect of ZnO-NP exposure level on total cells, macrophages, lymphocytes, neutrophils and eosinophils as well as significant positive effect of *Nrf2* deletion on total cells, macrophages and eosinophils. Single regression analyses showed significant positive trend with exposure level of ZnO-NPs for all of total cells, macrophages, lymphocytes, neutrophils and eosinophils in both of genotypes. There was no significant effect of ZnO-NP exposure level or *Nrf2* deletion on total protein level.

**Table 2:** The number of cells and protein in BALF from the mice at 14 days after exposure to zinc oxide nanoparticles by pharyngeal aspiration

		Amount of ZnO-NPs administered (μg)			Simple regression/Simple ordinal logistic regression	Multiple regression/Multiple ordinal logistic regression		
	Genotype	0	10	30	Effect of ZnO-NPs	Interaction of ZnO-NPs and Nrf2 deletion	Effect of ZnO-NPs	Effect of Nrf2 deletion
Total cells (× 10^4^)	Wild type *Nrf2*^−/−^	3 ± 1.7 3.7 ± 1.5	3.5 ± 2.0 6.3 ± 2.5	9.1 ± 2.9* 13.2 ± 6.0*	0.21(*p* < 0.0001) 0.32(*p* < 0.0001)	0.053(*p* = 0.12)	0.26(*p* < 0.0001)	1.3(*p* = 0.0041)
Macrophages (× 10^4^)	Wild type *Nrf2*^−/−^	3.1 ± 1.6 3.7 ± 1.5	3.5 ± 2.0 6.0 ± 2.3	8.4 ± 2.6* 12,0 ± 5.4*	0.19 (*p* < 0.0001) 0.28 (*p* < 0.0001)	0.047 (*p* = 0.14)	0.23(*p* < 0.0001)	1.1 (*p* = 0.0071)
Lymphocytes (× 10^4^)	Wild type *Nrf2*^−/−^	0.04 ± 0.04 0.04 ± 0.04	0.10 ± 0.12 0.26 ± 0.27	0.58 ± 0.32* 0.9 ± 0.7*	0.019 (*p* < 0.0001) 0.029 (*p* = 0.0002)	0.0051 (*p* = 0.15)	0.024(*p* < 0.0001)	0.081(*p* = 0.074)
Neutrophils (× 10^4^)	Wild type *Nrf2*^−/−^	0 0	0.01 ± 0.01 0.003 ± 0.004	0.02 ± 0.02 0.074 ± 0.07*	0.066 (*p* = 0.059) 0.16 (*p* = 0.002)	0.11 (*p* = 0.06)	0.11 (*p* = 0.0002)	0.70 (*p* = 0.28)
Eosinophils (× 10^4^)	Wild type *Nrf2*^−/−^	0 0	0.003 ± 0.005 0.013 ± 0.013*	0.04 ± 0.04* 0.16 ± 0.3*	0.16 (*p* = 0.0006) 0.24 (*p* = 0.0004)	0.016 (*p* = 0.76)	0.18 (*p* < 0.0001)	1.3 (*p* = 0.046)
Total protein (mg/ml)	Wild type *Nrf2*^−/−^	0.08 ± 0.02 0.08 ± 0.02	0.09 ± 0.03 0.08 ± 0.02	0.08 ± 0.02 0.07 ± 0.02	−0.00014 (*p* = 0.72) − 0.00028 (*p* = 0.35)	6.7 × 10^−5^ (*p* = 0.79)	0.00025 (*p* = 0.43)	−0.002(*p* = 0.67)

Mean ± SD. ^*^ *p* < 0.05, compared to the corresponding genotype control by Dunnett’s multiple comparison following ANOVA for the number of total cells, macrophages and lymphocytes, and total protein or by Steel multiple comparison following Kruskal Wallis nonparametric test for the number of neutrophils and eosinophils. Simple regression analysis in each genotype and multiple regression analysis was conducted in a model with interaction for total cells, macrophages, lymphocytes and total protein. As the interaction was not significant, multiple regression analysis was finally conducted in a model without interaction to estimate effect of ZnO-NPs or Nrf2 deletion. Simple ordinal logistic regression analysis in each genotype and multiple ordinal logistic regression analysis was conducted in a model with interaction for neutrophils or eosinophils. As the interaction was not significant, multiple ordinal logistic regression analysis was finally conducted in a model without interaction to estimate effect of ZnO-NPs or Nrf2 deletion

### Lung histopathological changes

**Fig. 1:**
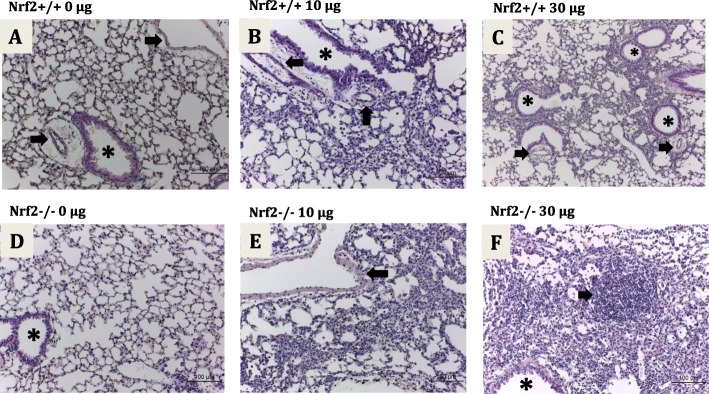
Representative micrographs of H&E-stained lung sections at post-exposure day 14. **a** Nrf2^+/+^ no ZnO-NPs, no inflammation is detected around blood vessels (arrows) or bronchioles (asterisk); **b** Nrf2^+/+^ 10 μg ZnO-NPs, bronchioles (asterisk) and vessels (arrows) are surrounded by a thin layer (1–5 cells thick) of inflammatory cells; **c** Nrf2^+/+^ 30 μg ZnO-NPs, bronchioles (asterisks) and vessels (arrows) are surrounded by a thick layer (> 5 cells thick) of inflammatory cells; **d** Nrf2^−/−^ no ZnO-NPs, no inflammation around bronchioles is detected (asterisk); **e** Nrf2^−/−^ 10 μg ZnO-NPs, vessels (arrow) are surrounded by a thin layer (1–5 cells thick) of inflammatory cells; **f** Nrf2^−/−^ 30 μg ZnO-NPs, bronchioles (asterisk) are surrounded by a thick layer (> 5 cells thick) of inflammatory cells with lymphoid follicle formation (arrow)

Hematoxylin and eosin (H&E)-stained lung sections showed pulmonary inflammation with peribronchial and perivascular infiltration of inflammatory cells in ZnO-NP exposed groups in both genotypes (Fig. 1). The lung inflammation scores (perivascular, peribronchial and total lung inflammation) were significantly higher in ZnO-NP exposed groups of both genotypes compared to the corresponding genotype controls (Table 3). Multiple ordinal logistic regression analysis with dependent variable of total inflammation score, perivascular inflammation score or peribronchial score showed neither significant interaction of genotype and ZnO-NP exposure level nor significant effect of genotype, but showed significant effect of ZnO-NP exposure level. No collagen deposition was detected in H&E-stained lung sections.

**Table 3:** Total inflammation score, perivascular inflammation score and peribronchial inflammation score from the mice at 14 days after exposure to zinc oxide nanoparticles by pharyngeal aspiration

		Amount of ZnO-NPs administered (μg)			Simple ordinal logistic regression	Multiple ordinal logistic regression		
	Genotype	0	10	30	Effect of ZnO-NPs	Interaction of ZnO-NPs and Nrf2 deletion	Effect of ZnO-NPs	Effect of Nrf2 deletion
Total inflammation score	Wild type *Nrf2*^−/−^	0 ± 0 0.13 ± 0.35	1.5 ± 0.9* 1.6 ± 0.9*	2.8 ± 0.5* 2.7 ± 0.5*	0.23(*p* < 0.0001) 0.20(*p* = 0.0002)	−0.060 (*p* = 0.27)	0.21(*p* < 0.0001)	0.078 (*p* = 0.89)
Perivascular inflammation	Wild type *Nrf2*^−/−^	0 ± 0 0.13 ± 0.35	1.4 ± 1.0* 1.4 ± 0.7*	2.7 ± 0.7* 2.6 ± 0.5*	0.23 (*p* < 0.0001) 0.41 (*p* = 0.0056)	−0.055 (*p* = 0.32)	0.24(*p* < 0.0001)	−0.033 (*p* = 0.96)
Peribronchial inflammation	Wild type *Nrf2*^−/−^	0 ± 0 0.13 ± 0.35	1.6 ± 1.0* 1.9 ± 1.1*	2.8 ± 0.4* 2.8 ± 0.5*	0.23(*p* < 0.0001) 0.17 (*p* = 0.0005)	−0.035 (*p* = 0.56)	0.20 (*p* < 0.0001)	0.33 (*p* = 0.59)

Mean ± SD. ^*^ *p* < 0.05, compared to the corresponding genotype control by Steel multiple comparison following Kruskal Wallis nonparametric test. Simple ordinal logistic regression analysis in each genotype and multiple ordinal logistic regression analysis in a model with interaction was conducted. As the interaction was not significant, multiple ordinal logistic regression analysis was finally conducted in a model without interaction to estimate effect of ZnO-NPs or Nrf2 deletion

### Changes in glutathione and MDA levels

Dunnett’s multiple comparison following ANOVA showed that exposure to ZnO-NPs at 30 μg per mouse significantly decreased GSSG or GSSG/GSH in *Nrf2*^−/−^ mice but not in wild type mice, as well as decreasing MDA in wild type mice but not in *Nrf2*^−/−^ mice (Table 4).

**Table 4:** Total GSH, GSSG, ratio of GSSG/total GSH and MDA in the lung of mice at 14 days after exposure to zinc oxide nanoparticles by pharyngeal aspiration

		Amount of ZnO-NPs administered (μg)			Simple regression	Multiple regression		
	Genotype	0	10	30	Effect of ZnO-NPs	Interaction of ZnO-NPs and Nrf2 deletion	Effect of ZnO-NPs	Effect of Nrf2 deletion
Total glutathione (GSH + GSSG, nmol / g lung tissue)	Wild type *Nrf2*^−/−^	47.9 ± 6.5 42.9 ± 10.4	41.4 ± 7.3 44.9 ± 7.5	45.4 ± 7.8 51.5 ± 5.2	−0.041 (*p* = 0.71) 0.29 (*p* = 0.032)	0.17 (*p* = 0.053)	0.11 (*p* = 0.22)	0.78 (*p* = 0.48)
Glutathione disulfide (GSSG, nmol / g lung tissue)	Wild type *Nrf2*^−/−^	5.8 ± 2.6 8.0 ± 2.2	5.1 ± 1.1 8.2 ± 2.0	6.2 ± 2.2 4.9 ± 2.0*	0.020 (*p* = 0.52) − 0.11 (*p* = 0.0036)	− 0.066 (*p* = 0.0057)	–	–
Ratio of GSSG/GSH (×10^−1^)	Wild type *Nrf2*^−/−^	1.48 ± 0.37 2.37 ± 0.67	1.45 ± 0.30 2.22 ± 4.41	1.62 ± 0.67 1.08 ± 0.54*	0.00052(*p* = 0.47) −0.0045 (*p* < 0.0001)	−0.0025 (*p* < 0.0001)	–	–
MDA (nmol / mg protein)	Wild type *Nrf2*^−/−^	2.34 ± 0.37 1.42 ± 0.18	2.27 ± 0.27 1.38 ± 0.47	1.27 ± 0.19* 1.97 ± 0.67	−0.038 (*p* < 0.0001) 0.020 (*p* = 0.019)	0.029 (*p* < 0.0001)	–	–

Mean ± SD. ^*^ *p* < 0.05compared to the corresponding genotype control by Dunnett’s multiple comparison following ANOVA. Simple regression analysis in each genotype and multiple regression analysis in a model with interaction was conducted. As the interaction was not significant for total glutathione, multiple regression in a model without interaction was finally conducted to estimate effect of ZnO-NPs and Nrf2 deletion

Multiple regression analyses showed significant interaction of ZnO-NP exposure level and *Nrf2* deletion for GSSG, GSSG/GSH and MDA, indicating different effect of ZnO-NP exposure level depending on the genotype. Single regression analyses showed that exposure to ZnO-NPs significantly decreased GSSG or GSSG/GSH in *Nrf2*^−/−^ mice but not in wild type mice, as well as increasing MDA in *Nrf2*^−/−^ mice while decreasing MDA in wild type mice. For total glutathione, multiple regression analyses showed neither significant interaction of ZnO-NP exposure level and *Nrf2* deletion, nor significant effect of ZnO-NP exposure level, nor significant effect of *Nrf2* deletion. Single regression analysis showed significant increase in total glutathione in *Nrf2*^−/−^ mice but not in wild type mice.

### Changes in lung mRNA levels

*(a) Antioxidant enzymes,* Dunnett’s multiple comparison following ANOVA showed that exposure to ZnO-NPs significantly increased mRNA expression of superoxide dismutase at 10 μg per mouse only in *Nrf2*^−/−^ mice and catalase at 30 μg per mouse in wild type mice, but did not change mRNA expression of *GcLc, GcLm, NQO1*, glutathione reductase or hemooxygenase-1 either in *Nrf2*^−/−^ mice or wild type mice (Table 5). Multiple regression analyses showed significant interaction of ZnO-NP exposure level and *Nrf2* deletion in glutathione reductase, indicating different effect of ZnO-NP exposure level depending on the genotype. Dunnett’s multiple comparison following ANOVA showed that exposure to ZnO-NPs at 30 μg per mouse significantly increased mRNA expression of metallothionein-1 and -2 in wild type mice but not in *Nrf2*^−/−^ mice. Multiple regression analyses showed significant interaction of ZnO-NP exposure level and *Nrf2* deletion for metallothionein-1, indicating different effect of ZnO-NP exposure level depending on the genotypes. Multiple regression analyses without interaction showed significant positive effect of ZnO-NP exposure level and *Nrf2* deletion on mRNA expression of metallothionein-2.

**Table 5:** Relative mRNA level for oxidative stress-related proteins to *β-actin*

		Amount of ZnO-NPs administered (μg)			Simple regression	Multiple regression		
	Genotype	0	10	30	Effect of ZnO-NPs	Interaction of ZnO-NPs and Nrf2 deletion	Effect of ZnO-NPs	Effect of Nrf2 deletion
*SOD1*	Wild type *Nrf2*^−/−^	1.5 ± 0.4 1.02 ± 0.3	1.8 ± 0.7 1.5 ± 0.3*	2.3 ± 0.9 1.2 ± 0.3	0.025(*p* = 0.027) 0.0023 (p = 0.68)	−0.011 (*p* = 0.083)	0.014(*p* = 0.028)	−0.31(*p* = 0.0003)
*CAT*	Wild type *Nrf2*^−/−^	2.4 ± 0.5 2.6 ± 0.7	1.9 ± 0.7 2.7 ± 1.0	3.2 ± 1.5(*p* = 0.042) 2.3 ± 0.8	0.030 (*p* = 0.063) −0.011 (*p* = 0.45)	−0.02 (*p* = 0.06)	0.012 (p = 0.28)	0.011 (*p* = 0.94)
*GcLc*	Wild type *Nrf2*^−/−^	1.6 ± 0.5 1.3 ± 0.4	1.4 ± 0.4 1.6 ± 0.5	1.9 ± 0.9 1.1 ± 0.2	0.011(*p* = 0.25) −0.011 (*p* = 0.14)	−0.011 (*p* = 0.082)	0.0016(*p* = 0.80)	−0.17(p = 0.032)
*GcLm*	Wild type *Nrf2*^−/−^	1.5 ± 0.6 1.3 ± 0.3	1.1 ± 0.3 1.3 ± 0.2	1.3 ± 0.5 1.0 ± 0.3	−0.0033 (*p* = 0.65) − 0.0095 (p = 0.48)	−0.0031 (*p* = 0.50)	− 0.0061 (*p* = 0.18)	−0.057(p = 0.32)
*NQO1*	Wild type *Nrf2*^−/−^	0.9 ± 0.5 0.7 ± 0.1	0.9 ± 0.8 0.9 ± 0.3	0.9 ± 0.8 0.6 ± 0.1	−0.00095 (*p* = 0.93) − 0.0045 (*p* = 0.23)	−0.0018 (*p* = 0.77)	− 0.0025 (*p* = 0.67)	−0.064(*p* = 0.39)
*GR*	Wild type *Nrf2*^−/−^	1.3 ± 0.7 2.1 ± 0.5	0.9 ± 0.3 1.8 ± 0.5	4.6 ± 6.7 1.7 ± 0.7	0.12(*p* = 0.040) −0.011 (*p* = 0.24)	−0.067 (*p* = 0.043)	–	–
*HO-1*	Wild type *Nrf2*^−/−^	7.5 ± 3.0 5.8 ± 2.5	7.1 ± 2.3 3.8 ± 1.6	6.8 ± 1.9 4.2 ± 1.7	−0.023 (*p* = 0.53) − 0.042 (p = 0.22)	0.0095 (*p* = 0.70)	− 0.031 (*p* = 0.21)	−1.25(*p* = 0.0002)
*MT-1*	Wild type *Nrf2*^−/−^	1.5 ± 0.9 1.9 ± 1.1	1.4 ± 0.6 2.2 ± 0.6	3.8 ± 2.5* 2.6 ± 0.9	0.081(*p* = 0.0017) −0.0033 (*p* = 0.83)	−0.042 (*p* = 0.0063)	–	–
*MT-2*	Wild type *Nrf2*^−/−^	1.9 ± 0.7 3.7 ± 1.6	1.9 ± 0.8 3.8 ± 1.4	3.9 ± 2.0* 4.1 ± 2.5	0.072 (*p* = 0.001) 0.013(*p* = 0.66)	−0.029 (*p* = 0.097)	0.046 (*p* = 0.012)	0.68(*p* = 0.0035)

Mean ± SD. ^*^ *p* < 0.05compared to the corresponding genotype control by Dunnett’s multiple comparison following ANOVA. Simple regression analysis in each genotype and multiple regression analysis in a model with interaction was conducted. As the interaction was not significant for *SOD1, CAT, GcLc, GcLm, NQO1, HO-1* or *MT-2*, multiple regression in a model without interaction was finally conducted to estimate effect of ZnO-NPs or Nrf2 deletion

*(b) Inflammatory cytokines;* Dunnett’s multiple comparison following ANOVA showed that exposure to ZnO-NPs at 30 μg per mouse increased mRNA expression of *TGF-β* and *IFN-γ* in wild type mice but not in *Nrf2*^−/−^ mice. Multiple regression analyses showed significant interaction of ZnO-NP exposure level and genotype for mRNA expression of *MIP-2*, indicating different effect of ZnO-NP exposure level depending on the genotype. Multiple regression analyses without interaction showed significant positive effect of ZnO-NP exposure level on *TGF-β* and significant positive effect of *Nrf2* deletion on *KC* and *IFN-γ*.

*(c) Fibrosis-related genes;* Dunnett’s multiple comparison following ANOVA showed that exposure to ZnO-NPs at 30 μg per mouse significantly increased mRNA expression of *MMP2* both in wild type mice and *Nrf2*^−/−^ mice (Table 6). Multiple regression analysis without interaction showed significant positive effect of ZnO-NP exposure level and *Nrf2* deletion on *MMP2*.

**Table 6:** Relative mRNA level for pro-inflammatory cytokines and proteins related with fibrosis to *β-actin*

		Amount of ZnO-NPs administered (μg)			Simple regression	Multiple regression		
	Genotype	0	10	30	Effect of ZnO-NPs	Interaction of ZnO-NPs and Nrf2 deletion	Effect of ZnO-NPs	Effect of Nrf2 deletion
*KC*	Wild type *Nrf2*^−/−^	1.0 ± 0.4 2.2 ± 1.3	1.4 ± 0.7 2.8 ± 1.0	1.2 ± 0.4 2.9 ± 1.4	0.0026 (*p* = 0.73) 0.023(*p* = 0.27)	0.010 (*p* = 0.30)	0.012(*p* = 0.25)	−0.71 (*p* < 0.0001)
*MIP-2*	Wild type *Nrf2*^−/−^	1.1 ± 0.6 2.1 ± 1.1	1.3 ± 0.8 2.4 ± 1.2	1.0 ± 0.3 3.9 ± 3.0	−0.0060 (*p* = 0.51) 0.064(*p* = 0.073)	0.035 (*p* = 0.028)	–	–
*IL-6*	Wild type *Nrf2*^−/−^	1.1 ± 0.4 1.1 ± 0.4	0.9 ± 0.3 1.1 ± 0.3	1.2 ± 0.6 1.5 ± 0.8	0.005(*p* = 0.48) 0.015(*p* = 0.14)	0.0049 (*p* = 0.39)	0.0090 (*p* = 0.11)	0.078 (*p* = 0.28)
*IL-1β*	Wild type *Nrf2*^−/−^	1.0 ± 0.7 0.9 ± 0.4	1.0 ± 0.6 1.1 ± 0.3	0.7 ± 0.3 1.1 ± 0.7	−0.0097 (*p* = 0.24) 0.005(*p* = 0.58)	0.0073 (*p* = 0.23)	−0.0034 (*p* = 0.58)	0.032 (*p* = 0.68)
*MCP-1*	Wild type *Nrf2*^−/−^	0.9 ± 0.7 0.7 ± 0.3	0.9 ± 1.0 0.6 ± 0.3	0.7 ± 0.4 0.9 ± 0.6	−0.0088 (*p* = 0.41) 0.0055 (*p* = 0.48)	0.0072 (*p* = 0.31)	−0.0027 (*p* = 0.70)	−0.038 (*p* = 0.67)
*TGF-β*	Wild type *Nrf2*^−/−^	1.1 ± 0.3 1.3 ± 0.2	1.1 ± 0.2 1.5 ± 0.7	1.6 ± 0.5* 1.4 ± 0.3	0.019 (*p* = 0.0011) 0.0028 (*p* = 0.70)	--0.0083 (*p* = 0.066)	0.012(*p* = 0.0098)	0.084 (*p* = 0.14)
*TNF-α*	Wild type *Nrf2*^−/−^	2.9 ± 1.0 2.9 ± 0.8	2.8 ± 0.6 2.6 ± 1.2	2.5 ± 0.7 3.1 ± 0.9	−0.012 (*p* = 0.30) 0.0089(*p* = 0.58)	0.010 (*p* = 0.28)	−0.0026 (*p* = 0.79)	0.092 (*p* = 0.43)
*IFN-γ*	Wild type *Nrf2*^−/−^	1.5 ± 0.6 3.5 ± 2.2	1.6 ± 0.5 4.0 ± 3.2	2.7 ± 0.9* 4.5 ± 3.9	0.042(*p* = 0.0004) 0.031 (*p* = 0.55)	--0.0053 (*p* = 0.82)	0.037 (*p* = 0.11)	1.1 (*p* = 0.0006)
*MMP2*	Wild type *Nrf2*^−/−^	0.7 ± 0.2 1.1 ± 0.4	0.7 ± 0.2 1.2 ± 0.4	1.5 ± 0.4* 1.9 ± 0.7*	0.027 (*p* = < 0.0001) 0.029 (*p* = 0.0022)	0.00092 (*p* = 0.84)	0.028 (*p* < 0.0001)	0.22 (*p* = 0.0002)

Mean ± SD. ^*^ *p* < 0.05compared to the corresponding genotype control by Dunnett’s multiple comparison following ANOVA. Simple regression analysis in each genotype and multiple regression analysis in a model with interaction was conducted. As the interaction was not significant for *KC, IL-6, IL-1β, MCP-1, TGF-β, TNF-α, IFN-γ* or *MMP2*, multiple regression in a model without interaction was finally conducted to estimate effect of ZnO-NPs or Nrf2 deletion

## Discussion

The results showed that a single exposure of murine lungs to ZnO-NPs increased the number of total cells, macrophages, lymphocytes, neutrophils and eosinophils in BALF both in wild type mice and *Nrf2*^−/−^ mice. Tests for interaction of multiple regression analyses or multiple ordinal logistic regression analysis suggest that effect of *Nrf2* deletion is additive to ZnO-NPs-induced increase in total cells, macrophages, lymphocytes or eosinophils while synergistic to ZnO-NPs-induced increase in neutrophils. The latter synergistic effect of *Nrf2* deletion might be explained by synergistic effect of *Nrf2* deletion on increase in mRNA expression of *MIP-2*, a neutrophil chemoattractant, which was revealed by quantitative real time PCR analysis. As *IFN-γ* is known to activate inflammatory cells, it is also possible to hypothesize that higher background level of *IFN-γ* mRNA induces higher background level of inflammatory cells in BALF of *Nrf2*^−/−^ mice. Given a recent study demonstrating that *Nrf2* negatively regulates expression of proinflammatory cytokines in macrophages through oxidative stress-independent pathway [42], the present study might show the role of *Nrf2* in regulation of proinflammatory response in alveolar macrophages, especially in neutrophil migration upon exposure to ZnO-NPs. ZnO-NPs-induced pulmonary inflammation in the present study is consistent with previous studies [9–13], but this is the first study to demonstrate the role of *Nrf2* in inflammatory response to ZnO-NP exposure in the lung of mice.

The significant up-regulation of *MMP2* mRNA expression in both genotypes can be linked to the observed pulmonary inflammation. Matrix metalloproteinase 2 (MMP2) is one of the matrix metalloproteinases (MMPs); a family of zinc-dependent endopeptidases that can degrade extracellular matrix and all its components including the basement membrane as well as key non-matrix mediators of lung injury, such as chemokines and cell surface receptors [43]. Gelatinases (MMP2 & MMP9) have been implicated in the pathogenesis of various lung diseases [44]. Enhanced activity of MMP2 or MMP9 is associated with bleomycin-induced pulmonary fibrosis [45], sustained lung inflammation following endotoxin exposure [46], interstitial fibrosis following repeated endotoxin exposure [47], lung injury following exposure to ozone [48] and subacute hyperoxia [49]. MMP2 and MMP9 were also described as essential for inflammatory cell infiltration and induction of airway hyperresponsiveness [50].

Regarding *Nrf2*/antioxidant response element (ARE)-dependent antioxidative enzymes, exposure to ZnO-NPs induced marginally increased mRNA level of superoxide dismutase 1 (*SOD1*) or catalase (*CAT*), but did not induce those of glutathione reductase (*GR*), glutamate-cysteine ligase catalytic subunit (*GcLc*)*,* glutamate-cysteine ligase regulatory subunit **(***GcLm*)*,* NAD(P) H quinone dehydrogenase (*NQO1*) or hemoxygenase-1 (*HO-1*) in wild type mice, suggesting weak potency of the examined ZnO-NPs in induction of ARE-dependent antioxidative enzymes. On the other hand, deletion of *Nrf2* reduced background level of mRNA expression of *SOD1*, *GcLc* and *HO-1*, suggesting the role of *Nrf2* in regulation on the background level of the above genes.

Exposure to ZnO-NPs increased mRNA expression of metallothionein-1 and -2 in wild type mice, being consistent with other studies [6, 8]. Deletion of *Nrf2* suppressed the ZnO-NP-induced increase in metallothionein-1 but increased the background level of metallothionein-2 expression. Metallothionein-1 and -2 are known to be regulated by metal response element (MRE), but recent studies show transcriptional induction of metallothionein-1 thorough *Nrf2*/ARE pathway [51–53], which might partly explain the suppression of ZnO-NP-induced increase in metallothionein-1. Metallothioneins are cysteine-rich and heavy metal-binding proteins known to contribute to a wide range of protective cellular responses. Their functions include detoxification of heavy metals, homeostasis of essential metals including zinc, protection against oxidants, reactive oxygen species, oxidative stress, DNA damage and apoptosis, as well as increasing cell proliferation [54]. Importantly, we found higher *MT-2* mRNA expression in *Nrf2*^−/−^ mice, which might represent a compensatory augmentation associated with deletion of *Nrf2*; consistent with the results of a study that showed compensatory increase in mRNA levels of *MTs* (among other antioxidant defenses) in *GcLm*-null mice exposed to ozone [55].

On the other hand, effects of *Nrf2* deletion or exposure to ZnO-NPs on oxidative stress markers GSSG/GSH or MDA are inconsistent, as shown by opposite effects of *Nrf2* deletion on background level of GSSG/GSH or MDA between wild type and *Nrf2*^−/−^ mice, as well as opposite trends of change with ZnO-NP exposure level between GSSG/GSH and MDA in *Nrf2*^−/−^ mice. Contrary to the present result in wild type mice, increase in MDA [16] and total hydroxyoctadecadienoic acid (tHODE), another marker of lipid peroxidation [15] was detected in BALF of rats exposed to ZnO-NPs, but it is also reported that ZnO-NPs has antioxidative effect [56–58]. Further studies are needed to investigate the effect of ZnO-NPs on the antioxidative system in vivo in relation with synthetic method of ZnO-NPs.

**Fig. 2:**
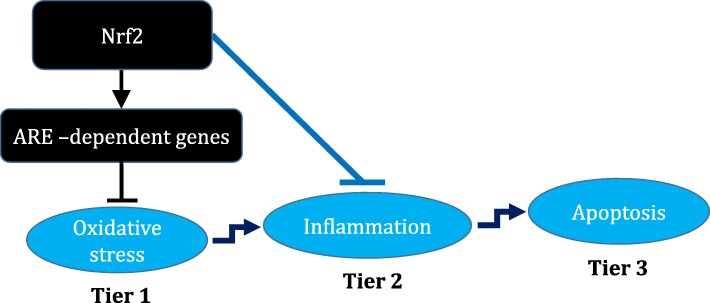
Schema for role of Nrf2 in suppressing oxidative stress and inflammatory response in relation to hierarchical oxidative stress hypothesis

The present study does not provide a typical model supporting the hierarchical oxidative stress hypothesis, as it demonstrated minimal induction of *Nrf2*/antioxidant response element (ARE)-dependent antioxidative enzymes and inconsistent induction of oxidative stress markers. However, the study demonstrated infiltration of inflammatory cells in the lung accompanied by marginal increase in expression of *MIP-2* in the lung of *Nrf2*^−/−^ mice exposed to ZnO-NPs, suggesting direct effect of Nrf2 on regulation of inflammatory cytokine genes in lung cells (Fig. 2).

In contrast to BALF results, inflammation scores based on histopathological examination did not show significant effect of *Nrf2* deletion. This might be due to the limitation of semi-quantification in inflammation scoring.

In the present study we chose male mice, as it was difficult to estimate how estrous cycle affected the biological indices and how we needed to adjust estrous cycle in administration of ZnO-NPs or dissection. As shown by a recent study [59], it is possible to observe gender difference in response to ZnO-NPs. Further studies on female mice are needed to investigate possible gender difference in susceptibility to ZnO-NP exposure.

In conclusions, deletion of *Nrf2* enhanced neutrophil infiltration in the BALF of mice exposed to ZnO-NPs, as well as enhancing the background level of the number of inflammatory cells in the BALF. Upregulation of *MIP-2* and *IFN-γ* after deletion of *Nrf2* might explain the above infiltration of BALF inflammatory cells. On the other hand, exposure to ZnO-NPs did not induce or only marginally induced *Nrf2*-dependent antioxidant enzymes and effect of *Nrf2* deletion on oxidative stress was inconsistent. The study showed protective role of Nrf2 in inflammation in the lung of mice exposed to ZnO-NPs, but it was difficult to fully explain this role by suppression of oxidative stress. Further studies are needed to clarify the mechanism of how Nrf2 negatively regulate migration of neutrophil or other inflammatory cells in the lung.

## Supplementary information

**Additional file 1: Table S1.** Sequence of primers used in real-time PCR.

## Data Availability

The authors declare that the data supporting the findings of this study are available within the paper and its supplementary information files.

## References

[1] Savolainen K, Pylkkanen L, Norppa H, Falck G, Lindberg H, Tuomi T, Vippola M, Alenius H, Hameri K, Koivisto J, Brouwer D, Mark D, Bard D, Berges M, Jankowska E, Posniak M, Farmer P, Singh R, Krombach F, Bihari P, Kasper G, Seipenbusch M (2010). Nanotechnologies, engineered nanomaterials and occupational health and safety - a review. Saf Sci.

[2] Huang YW, Wu CH, Aronstam RS (2010). Toxicity of transition metal oxide nanoparticles: recent insights from in vitro studies. Materials (Basel).

[3] Chang YN, Guo H, Li J, Song Y, Zhang M, Jin J, Xing G, Zhao Y (2013). Adjusting the balance between effective loading and vector migration of macrophage vehicles to deliver nanoparticles. PLoS One.

[4] Krug HF, Wick P (2011). Nanotoxicology: an interdisciplinary challenge. Angew Chem Int Ed Engl.

[5] Baek M, Chung HE, Yu J, Lee JA, Kim TH, Oh JM, Lee WJ, Paek SM, Lee JK, Jeong J, Choy JH, Choi SJ (2012). Pharmacokinetics, tissue distribution, and excretion of zinc oxide nanoparticles. Int J Nanomedicine.

[6] Sahu D, Kannan GM, Vijayaraghavan R, Anand T, Khanum F (2013). Nanosized zinc oxide induces toxicity in human lung cells. ISRN Toxicol.

[7] Xia T, Kovochich M, Liong M, Madler L, Gilbert B, Shi H, Yeh JI, Zink JI, Nel AE (2008). Comparison of the mechanism of toxicity of zinc oxide and cerium oxide nanoparticles based on dissolution and oxidative stress properties. ACS Nano.

[8] Zhang J, Song W, Guo J, Zhang J, Sun Z, Ding F, Gao M (2012). Toxic effect of different ZnO particles on mouse alveolar macrophages. J Hazard Mater.

[9] Cho WS, Duffin R, Howie SE, Scotton CJ, Wallace WA, Macnee W, Bradley M, Megson IL, Donaldson K (2011). Progressive severe lung injury by zinc oxide nanoparticles; the role of Zn2+ dissolution inside lysosomes. Part Fibre Toxicol.

[10] Cho WS, Duffin R, Poland CA, Howie SE, MacNee W, Bradley M, Megson IL, Donaldson K (2010). Metal oxide nanoparticles induce unique inflammatory footprints in the lung: important implications for nanoparticle testing. Environ Health Perspect.

[11] Chang H, Ho CC, Yang CS, Chang WH, Tsai MH, Tsai HT, Lin P (2013). Involvement of MyD88 in zinc oxide nanoparticle-induced lung inflammation. Exp Toxicol Pathol.

[12] Adamcakova-Dodd A, Stebounova LV, Kim JS, Vorrink SU, Ault AP, O'Shaughnessy PT, Grassian VH, Thorne PS (2014). Toxicity assessment of zinc oxide nanoparticles using sub-acute and sub-chronic murine inhalation models. Part Fibre Toxicol.

[13] Warheit DB, Sayes CM, Reed KL (2009). Nanoscale and fine zinc oxide particles: can in vitro assays accurately forecast lung hazards following inhalation exposures?. Environ Sci Technol.

[14] Akhtar MJ, Ahamed M, Kumar S, Khan MM, Ahmad J, Alrokayan SA (2012). Zinc oxide nanoparticles selectively induce apoptosis in human cancer cells through reactive oxygen species. Int J Nanomedicine.

[15] Fukui H, Horie M, Endoh S, Kato H, Fujita K, Nishio K, Komaba LK, Maru J, Miyauhi A, Nakamura A, Kinugasa S, Yoshida Y, Hagihara Y, Iwahashi H (2012). Association of zinc ion release and oxidative stress induced by intratracheal instillation of ZnO nanoparticles to rat lung. Chem Biol Interact.

[16] Liu H, Yang D, Yang H, Zhang H, Zhang W, Fang Y, Lin Z, Tian L, Lin B, Yan J, Xi Z (2013). Comparative study of respiratory tract immune toxicity induced by three sterilisation nanoparticles: silver, zinc oxide and titanium dioxide. J Hazard Mater.

[17] Yu KN, Yoon TJ, Minai-Tehrani A, Kim JE, Park SJ, Jeong MS, Ha SW, Lee JK, Kim JS, Cho MH (2013). Zinc oxide nanoparticle induced autophagic cell death and mitochondrial damage via reactive oxygen species generation. Toxicol Vitro.

[18] Song W, Zhang J, Guo J, Zhang J, Ding F, Li L, Sun Z (2010). Role of the dissolved zinc ion and reactive oxygen species in cytotoxicity of ZnO nanoparticles. Toxicol Lett.

[19] Shen C, James SA, de Jonge MD, Turney TW, Wright PF, Feltis BN (2013). Relating cytotoxicity, zinc ions, and reactive oxygen in ZnO nanoparticle-exposed human immune cells. Toxicol Sci.

[20] Kao YY, Chen YC, Cheng TJ, Chiung YM, Liu PS (2012). Zinc oxide nanoparticles interfere with zinc ion homeostasis to cause cytotoxicity. Toxicol Sci.

[21] Xia T, Zhao Y, Sager T, George S, Pokhrel S, Li N, Schoenfeld D, Meng H, Lin S, Wang X, Wang M, Ji Z, Zink JI, Madler L, Castranova V, Lin S, Nel AE (2011). Decreased dissolution of ZnO by iron doping yields nanoparticles with reduced toxicity in the rodent lung and zebrafish embryos. ACS Nano.

[22] Cho WS, Duffin R, Poland CA, Duschl A, Oostingh GJ, Macnee W, Bradley M, Megson IL, Donaldson K (2012). Differential pro-inflammatory effects of metal oxide nanoparticles and their soluble ions in vitro and in vivo; zinc and copper nanoparticles, but not their ions, recruit eosinophils to the lungs. Nanotoxicology.

[23] Lin WS, Xu Y, Huang CC, Ma YF, Shannon KB, Chen DR, Huang YW (2009). Toxicity of nano- and micro-sized ZnO particles in human lung epithelial cells. J Nanopart Res.

[24] De Stefano D, Carnuccio R, Maiuri MC (2012). Nanomaterials toxicity and cell death modalities. J Drug Deliv.

[25] Nel A. (2006). Toxic Potential of Materials at the Nanolevel. Science.

[26] Kim J, Cha YN, Surh YJ (2010). A protective role of nuclear factor-erythroid 2-related factor-2 (Nrf2) in inflammatory disorders. Mutat Res.

[27] Marzec JM, Christie JD, Reddy SP, Jedlicka AE, Vuong H, Lanken PN, Aplenc R, Yamamoto T, Yamamoto M, Cho HY, Kleeberger SR (2007). Functional polymorphisms in the transcription factor NRF2 in humans increase the risk of acute lung injury. FASEB J.

[28] Cho HY, Kleeberger SR (2010). Nrf2 protects against airway disorders. Toxicol Appl Pharmacol.

[29] Chan K, Kan YW (1999). Nrf2 is essential for protection against acute pulmonary injury in mice. Proc Natl Acad Sci U S A.

[30] Rangasamy T, Guo J, Mitzner WA, Roman J, Singh A, Fryer AD, Yamamoto M, Kensler TW, Tuder RM, Georas SN, Biswal S (2005). Disruption of Nrf2 enhances susceptibility to severe airway inflammation and asthma in mice. J Exp Med.

[31] Rangasamy T, Cho CY, Thimmulappa RK, Zhen L, Srisuma SS, Kensler TW, Yamamoto M, Petrache I, Tuder RM, Biswal S (2004). Genetic ablation of Nrf2 enhances susceptibility to cigarette smoke-induced emphysema in mice. J Clin Invest.

[32] Kikuchi N, Ishii Y, Morishima Y, Yageta Y, Haraguchi N, Itoh K, Yamamoto M, Hizawa N (2010). Nrf2 protects against pulmonary fibrosis by regulating the lung oxidant level and Th1/Th2 balance. Respir Res.

[33] Cho HY, Gladwell W, Yamamoto M, Kleeberger SR (2013). Exacerbated airway toxicity of environmental oxidant ozone in mice deficient in Nrf2. Oxidative Med Cell Longev.

[34] Li YJ, Takizawa H, Azuma A, Kohyama T, Yamauchi Y, Takahashi S, Yamamoto M, Kawada T, Kudoh S, Sugawara I (2008). Disruption of Nrf2 enhances susceptibility to airway inflammatory responses induced by low-dose diesel exhaust particles in mice. Clin Immunol.

[35] Porter D, Sriram K, Wolfarth M, Jefferson A, Schwegler-Berry D, Andrew M, Castranova V (2008). A biocompatible medium for nanoparticle dispersion. Nanotoxicology.

[36] Wu W, Ichihara G, Suzuki Y, Izuoka K, Oikawa-Tada S, Chang J, Sakai K, Miyazawa K, Porter D, Castranova V, Kawaguchi M, Ichihara S (2014). Dispersion method for safety research on manufactured nanomaterials. Ind Health.

[37] Itoh K, Chiba T, Takahashi S, Ishii T, Igarashi K, Katoh Y, Oyake T, Hayashi N, Satoh K, Hatayama I, Yamamoto M, Nabeshima Y (1997). An Nrf2/small Maf heterodimer mediates the induction of phase II detoxifying enzyme genes through antioxidant response elements. Biochem Biophys Res Commun.

[38] Kinaret P, Ilves M, Fortino V, Rydman E, Karisola P, Lahde A, Koivisto J, Jokiniemi J, Wolff H, Savolainen K, Greco D, Alenius H (2017). Inhalation and Oropharyngeal aspiration exposure to rod-like carbon nanotubes induce similar airway inflammation and biological responses in mouse lungs. ACS Nano.

[39] Scanlan CR, Wilkins RL, Stoller JK (1999). *Egan's Fundamental of Respiratory Care*.

[40] Braber S, Henricks PA, Nijkamp FP, Kraneveld AD, Folkerts G (2010). Inflammatory changes in the airways of mice caused by cigarette smoke exposure are only partially reversed after smoking cessation. Respir Res.

[41] Kwak YG, Song CH, Yi HK, Hwang PH, Kim JS, Lee KS, Lee YC (2003). Involvement of PTEN in airway hyperresponsiveness and inflammation in bronchial asthma. J Clin Invest.

[42] Kobayashi EH, Suzuki T, Funayama R, Nagashima T, Hayashi M, Sekine H, Tanaka N, Moriguchi T, Motohashi H, Nakayama K, Yamamoto M (2016). Nrf2 suppresses macrophage inflammatory response by blocking proinflammatory cytokine transcription. Nat Commun.

[43] Davey A, DF MA, O'Kane CM (2011). Matrix metalloproteinases in acute lung injury: mediators of injury and drivers of repair. Eur Respir J.

[44] Chakrabarti S, Patel KD (2005). Matrix metalloproteinase-2 (MMP-2) and MMP-9 in pulmonary pathology. Exp Lung Res.

[45] Kim JY, Choeng HC, Ahn C, Cho SH (2009). Early and late changes of MMP-2 and MMP-9 in bleomycin-induced pulmonary fibrosis. Yonsei Med J.

[46] D'Ortho MP, Jarreau PH, Delacourt C, Macquin-Mavier I, Levame M, Pezet S, Harf A, Lafuma C (1994). Matrix metalloproteinase and elastase activities in LPS-induced acute lung injury in Guinea pigs. Am J Phys.

[47] Corbel M, Theret N, Caulet-Maugendre S, Germain N, Lagente V, Clement B, Boichot E (2001). Repeated endotoxin exposure induces interstitial fibrosis associated with enhanced gelatinase (MMP-2 and MMP-9) activity. Inflamm Res.

[48] Sunil VR, Patel-Vayas K, Shen J, Laskin JD, Laskin DL (2012). Classical and alternative macrophage activation in the lung following ozone-induced oxidative stress. Toxicol Appl Pharmacol.

[49] Pardo A, Barrios R, Maldonado V, Melendez J, Perez J, Ruiz V, Segura-Valdez L, Sznajder JI, Selman M (1998). Gelatinases a and B are up-regulated in rat lungs by subacute hyperoxia: pathogenetic implications. Am J Pathol.

[50] Kumagai K, Ohno I, Okada S, Ohkawara Y, Suzuki K, Shinya T, Nagase H, Iwata K, Shirato K (1999). Inhibition of matrix metalloproteinases prevents allergen-induced airway inflammation in a murine model of asthma. J Immunol.

[51] Fujie Tomoya, Murakami Masaki, Yoshida Eiko, Yasuike Shuji, Kimura Tomoki, Fujiwara Yasuyuki, Yamamoto Chika, Kaji Toshiyuki (2016). Transcriptional Induction of Metallothionein by Tris(pentafluorophenyl)stibane in Cultured Bovine Aortic Endothelial Cells. International Journal of Molecular Sciences.

[52] Fujie T, Segawa Y, Yoshida E, Kimura T, Fujiwara Y, Yamamoto C, Satoh M, Naka H, Kaji T (2016). Induction of metallothionein isoforms by copper diethyldithiocarbamate in cultured vascular endothelial cells. J Toxicol Sci.

[53] Fujie T, Takenaka F, Yoshida E, Yasuike S, Fujiwara Y, Shinkai Y, Kumagai Y, Yamamoto C, Kaji T (2019). Possible mechanisms underlying transcriptional induction of metallothionein isoforms by tris (pentafluorophenyl) stibane, tris (pentafluorophenyl) arsane, and tris (pentafluorophenyl) phosphane in cultured bovine aortic endothelial cells. J Toxicol Sci.

[54] Thirumoorthy N, Shyam Sunder A, Manisenthil Kumar K, Senthil Kumar M, Ganesh G, Chatterjee M (2011). A review of metallothionein isoforms and their role in pathophysiology. World J Surg Oncol.

[55] Johansson E, Wesselkamper SC, Shertzer HG, Leikauf GD, Dalton TP, Chen Y (2010). Glutathione deficient C57BL/6J mice are not sensitized to ozone-induced lung injury. Biochem Biophys Res Commun.

[56] Murali M, Mahendra C, Nagabhushan, Rajashekar N, Sudarshana MS, Raveesha KA, Amruthesh KN (2017). Antibacterial and antioxidant properties of biosynthesized zinc oxide nanoparticles from Ceropegia candelabrum L. - an endemic species. Spectrochim Acta A Mol Biomol Spectrosc.

[57] Nagajyothi PC, Cha SJ, Yang IJ, Sreekanth TV, Kim KJ, Shin HM (2015). Antioxidant and anti-inflammatory activities of zinc oxide nanoparticles synthesized using Polygala tenuifolia root extract. J Photochem Photobiol B.

[58] Shoae-Hagh P, Rahimifard M, Navaei-Nigjeh M, Baeeri M, Gholami M, Mohammadirad A, Abdollahi M (2014). Zinc oxide nanoparticles reduce apoptosis and oxidative stress values in isolated rat pancreatic islets. Biol Trace Elem Res.

[59] Shvedova AA, Kisin ER, Yanamala N, Farcas MT, Menas AL, Williams A, Fournier PM, Reynolds JS, Gutkin DW, Star A, Reiner RS, Halappanavar S, Kagan VE (2016). Gender differences in murine pulmonary responses elicited by cellulose nanocrystals. Part Fibre Toxicol.

