# Supplementary material for: Role of Nrf2 in inflammatory response in lung of mice exposed to zinc oxide nanoparticles
Source: Part Fibre Toxicol. 2019 Dec 16;16:47. doi: 10.1186/s12989-019-0328-y (PMC6915997; doi:10.1186/s12989-019-0328-y)
Supplement: Supplementary file 1 — Additional file 1: Table S1. Sequence of primers used in real-time PCR. [file 12989_2019_328_MOESM1_ESM.docx]

**Table S1. Sequence of primers used in real-time PCR.**

| Gene symbol (GeneBank accession No.) |  | Sequence (5'--->3') |
| --- | --- | --- |
| *SOD1* (NM_011434.1) | F  R | CAGGACCTCATTTTAATCCTCAC  TGCCCAGGTCTCCAACAT |
| *CAT*  (NM_009804.2) | F  R | GGCAGTCTATTGCAAGTTCCAT  ATCCGGATCTTCCTGAGCA |
| *GcLc* (NM_010295.2) | F  R | AGATGATAGAACACGGGAGGAG  TGATCCTAAAGCGATTGTTCTTC |
| *GcLm* (NM_008129.3) | F  R | TGGAGCAGCTGTATCAGTGG  CAAAGGCAGTCAAATCTGGTG |
| *NQO1* (NM_008706.5) | F  R | AGCGTTCGGTATTACGATCC  AGTACAATCAGGGCTCTTCTCG |
| *GR* (NM_010344.4) | F  R | GTTCCTCACGAGAGCCAGAT  TCCAGCTGAAAGAAGCCATC |
| *HO-1* (NM_010442.2) | F  R | AGGCTAAGACCGCCTTCCT  TGTGTTCCTCTGTCAGCATCA |
| *MT-1*(NM_013602.3) | F  R | CAAGTGCACCTCCTGCAA  TTCGTCACATCAGGCACAG |
| *MT-2* (NM_008630.2) | F  R | CATGGACCCCAACTGCTC  AGCAGGAGCAGCAGCTTT |
| *KC (*NM_008176.3) | F  R | ACTCCAACACAGCACCATGA  TGGTCTGCAGGCACTGAC |
| *MIP-2 (*NM_009140.2) | F  R | AAAATCATCCAAAAGATACTGAAC AA  TTCTCTTTGGTTCTTCCGTTG |
| *IL-6* ([NM_031168.2](https://qpcr.probefinder.com/showsequence.jsp;jsessionid=53ED45E57F268A5EF45F32806929489A.worker13?seqNo=266805642)) | F  R | GCTACCAAACTGGATATAATCAGGA  CCAGGTAGCTATGGTACTCCAGAA |
| *IL-1β* ([NM_008361.4](https://qpcr.probefinder.com/showsequence.jsp;jsessionid=919AAAC1EFF6328D1CE6360A59114BA1.worker13?seqNo=32895469)) | F  R | AGCTTCAGGCAGGCAGTATC  GTCACAGAGGATGGGCTCTT |
| *MCP-1* (NM_011333.3) | F  R | CATCCACGTGTTGGCTCA  GATCATCTTGCTGGTGAATGAGT |
| *TGF-β* ([NM_011577.1](https://qpcr.probefinder.com/MainServlet.do)) | F  R | TCAGACATTCGGGAAGCAGT  ACGCCAGGAATTGTTGCTAT |
| *TNF-α* ([NM_013693.2](https://qpcr.probefinder.com/MainServlet.do)) | F  R | TCTTCTCATTCCTGCTTGTGG  GGTCTGGGCCATAGAACTGA |
| *IFN-γ* ([NM_008337.3](https://qpcr.probefinder.com/MainServlet.do)) | F  R | ATCTGGAGGAACTGGCAAAA  TTCAAGACTTCAAAGAGTCTGAGGTA |
| *MMP2* (NM_008610.2) | F  R | GGAGAAGGCTGTGTTCTTCG  AGGCTGGTCAGTGGCTTG |
| *β-actin* (NM_007393.3) | F  R | AAGGCCAACCGTGAAAAGAT  GTGGTACGACCAGAGGCATAC |
